# Unattractive people are unaware of their (un)attractiveness

**DOI:** 10.1111/sjop.12631

**Published:** 2020-03-11

**Authors:** Tobias Greitemeyer

**Affiliations:** ^1^ University of Innsbruck Innsbruck Austria

**Keywords:** Physical attractiveness, self‐serving bias, above average effect, Dunning‐Kruger effect

## Abstract

Past research has shown that how people rate their physical attractiveness is only moderately correlated with how they are rated by others, suggesting that at least some people have little insight into their true level of attractiveness. The present research tests the hypothesis that unattractive people are not aware of their unattractiveness. In fact, six studies (overall *N *=* *1,180) showed that unattractive participants considerably overestimated their attractiveness compared to ratings by strangers. In contrast, attractive participants were more accurate. If anything, they underestimated their attractiveness. It was also examined why unattractive people overestimate their attractiveness. As expected, unattractive participants differentiated less between attractive and unattractive stimulus persons than did attractive participants. They were also more likely than attractive participants to select unattractive stimulus persons to compare themselves to. However, these tendencies did not account for why unattractive participants overestimated their attractiveness, nor did affirming participant’s self‐worth. Limitations and avenues for future research are discussed.

## Introduction

The physical attractiveness^1^ of a person has important implications for how this person is treated by others. Attractive people are more likely to receive help (Benson, Karabenick & Lerner, 1976) and less likely to be punished (Berkowitz & Frodi, 1979), their performance is rated more favorably (Landy & Sigall, 1974), and they are more likely to be desired as a romantic partner (Walster, Aronson, Abrahams & Rottman, 1966). Contrary to the maxim “beauty is in the eye of the beholder,” there is generally high agreement about who is attractive and who is not (for meta‐analyses, Feingold, 1992; Langlois, Kalakanis, Rubenstein, Larson, Hallam & Smoot, 2000). Given the importance of physical attractiveness for people’s daily life and high agreement in attractiveness judgments, one may assume that people are well aware of whether they are attractive or not. However, abundant evidence has shown that self‐assessed attractiveness (in the following, subjective attractiveness) and how a person is rated by others (in the following, objective attractiveness) are only moderately related. In a meta‐analysis of 21 studies (Feingold, 1992), the correlation between subjective and objective attractiveness was *r *=* *0.24. To sum up, others mostly agree about whether a person is attractive or not, but this person does not necessarily agree.

In the present research, the idea is examined that those who are objectively unattractive overestimate their attractiveness and thus maintain unrealistically positive self‐views. In contrast, attractive people should be more accurate in their self‐rated attractiveness. A further purpose was to establish what psychological processes account for the tendency that unattractive people overestimate their attractiveness. As will be seen, whereas all studies consistently show that unattractive participants overestimate their attractiveness, the operating mechanisms are rather elusive.

### Overly positive self‐perceptions

Hundreds of studies have shown that people are prone to favorable self‐views. For example, people tend to make favorable comparisons to their own past selves (Wilson & Ross, 2000) and report that they will achieve more in the future compared to what they had in the past (Johnson, 2009). Other research has shown that people are likely to claim that they possess more positive qualities (e.g., being a better driver, more athletic, more intelligent) and fewer character flaws (e.g., engaging in immoral behaviors) than the average person (above‐average effect; Alicke, Klotz, Breitenbecher, Yurak & Vredenburg, 1995; Chambers & Windschitl, 2004; Logg, Haran & Moore, 2018). This tendency to perceive oneself as being better than the average peers is particularly pronounced when it comes to traits that are highly important, whereas it is less pronounced when it comes to traits of less importance (Brown, 2012). Interestingly, people tend to believe that they are less prone than others to be positively biased in their self‐perceptions (Pronin, Lin & Ross, 2002). Overall, at least in Western cultures, most people think highly of themselves compared with not only other people but also objective standards (for a review, Zell & Krizan, 2014).

Likewise, people typically maintain unrealistic positive perceptions of their own body size (Mazzurega, Marisa, Zampini & Pavani, in press) and most people rate themselves more attractive compared to how they are rated by strangers (e.g., Murstein & Christy, 1976; Springer, Wiltfang, Kowalski *et al.*, 2012; Yoder, Ault & Mathews, 2017). However, the tendency to overestimate one’s attractiveness cannot necessarily account for the relatively small correlation between people’s subjective and objective attractiveness. If everyone overestimates their level of attractiveness compared to how they are perceived by others to the same degree, then a perfect correlation would result. This means that some people more than others have to overestimate their attractiveness.

### Who is most prone to flawed self‐assessments?

A seminal investigation (Kruger & Dunning, 1999) assessed participant’s performance in a competence task. Afterwards, participants were asked how they perceived their performance. Across different competence domains (e.g., logical reasoning and grammar skills), incompetent participants had little insights into how competent they actually were. In fact, they dramatically overestimated how they performed in the test and they had unrealistically positive perceptions of their general ability. In contrast, relatively competent participants did not overestimate their test performance and general ability. If anything, they *under*estimated them. In the end, self‐rated competence by objectively incompetent and competent participants hardly differed, although there were large differences in actual abilities. This basic finding that the incompetent overestimate their abilities – termed the Dunning‐Kruger effect – has been replicated in dozens of studies (for a review, Dunning, 2011). Critics (Krueger & Mueller, 2002) argued that the Dunning‐Kruger effect is simply a form of a statistical artifact driven by regression‐to‐the‐mean. However, some of the findings in the initial report (Studies 3 and 4, Kruger & Dunning, 1999) as well as subsequent research (e.g., Ehrlinger, Johnson, Banner, Dunning & Kruger, 2008) speak against the regression effect.

Overall, there is overwhelming evidence that incompetent people fail to recognize their own incompetence. The main underlying mechanism why incompetent people overestimate their competence is the inability to recognize that they perform poorly (Kruger & Dunning, 1999). That is, it is not that the overestimation is driven by a desire to have favorable self‐views, they simply lack the skills that are needed to evaluate competence. In fact, incompetent people have deficient general metacognitive abilities as they not only fail to recognize their own faults, but are also less capable to recognize if others are failing or shining. When incompetent participant's metacognitive skills were improved, then the accuracy of how they perceive their own competence was also improved. Ironically, after becoming more competent, they recognized their own incompetence. In contrast, competent people have these metacognitive abilities, but they fall prey to a false‐consensus effect, in that they underestimate the skills of their peers because solving the test is easy for them and thus they wrongly assume that others also easily succeed. As a consequence, they underestimate their own performance.

### Why could unattractive people overestimate their attractiveness?

Analogous to the Dunning‐Kruger effect, the present research addresses the idea that objectively unattractive individuals are not aware of their own (un)attractiveness. Relative to how they are rated by others, unattractive people should overestimate their attractiveness. Attractive people, in contrast, should not over‐ but may even underestimate their attractiveness. Previous research into whether people have accurate or inaccurate views of their own attractiveness provided mixed evidence, with some research suggesting that people are mostly aware of how attractive they are seen by others (Marcus & Miller, 2003) and other findings suggesting that there is little consensus between self‐assessed attractiveness and ratings by others and that unattractive people in particular overestimate their attractiveness (Gurman & Balban, 1990). Hence, I deemed it important to provide a comprehensive test of the idea that unattractive more than attractive people overestimate their attractiveness.

The present research also addressed why unattractive people overestimate their attractiveness. As just noted, incompetent people lack the metacognitive skills that are needed to recognize their own incompetence. Relatedly, it was examined whether unattractive people would have less general insight into who is attractive and who is not. It might be that unattractive people have a different beauty ideal than do attractive people and thus would not only overestimate their own attractiveness, but also perceive unattractive others to be more attractive compared to how these are rated by attractive people. It was therefore investigated if unattractive participants overestimate their own attractiveness the less they differentiate between attractive and unattractive stimulus persons.

Another underlying mechanism might be that unattractive and attractive people select different comparison targets. Previous research has shown that people are affected in their attractiveness ratings by prior exposure to attractive or unattractive stimulus persons. In one study (Kenrick & Gutierres, 1980), a photo of an average looking female was rated less attractive after male raters had been exposed to highly attractive female stimulus persons. Other research (Cash, Cash & Butters, 1983) showed that self‐evaluations of physical attractiveness are also affected by contextual contrast effects. After exposure to attractive stimulus persons (compared to exposure to unattractive stimulus persons), female participants rated their own attractiveness lower. Subsequent research (Thornton & Moore, 1993) documented that not only female judgments but also male judgements of their own attractiveness are lower after exposure to attractive stimulus persons and higher after exposure to unattractive stimulus persons. Given that people tend to compare themselves with others who they feel are similar (Wood, 1989) and who they view as relevant to the self (Lockwood & Kunda, 1997), unattractive people may compare themselves with others that are unattractive, whereas attractive people compare themselves with attractive others. As a consequence, both could come to the conclusion that their attractiveness level is similar to most others, which results in unattractive people overestimating their attractiveness and attractive people underestimating it.

A further mechanism that was examined was whether unattractive people truly believe their reported attractiveness level or whether they know that they are unattractive. It is almost a truism that everyone has the wish to perceive oneself positively (e.g., to be attractive). If this favorable view is threatened, defensive responses often take place that directly reduce the threat. If, however, the self is protected through the affirmation of alternative sources of self‐integrity, defensive biases are less likely to occur. In line with self‐affirmation theory (Steele, 1988), it was examined whether unattractive people would perceive themselves more accurately after they had affirmed aspects of their self that were unconnected to their appearance. Moreover, previous research (Bollich, Rogers & Vazire, 2015) has shown that most people often are aware of whether their self‐perceptions are biased. Compared to an objective criterion, participants with positive biases admitted to be positively biased and participants with negative biases accurately reported on their negative biases. These findings suggest that even when people have biased self‐perceptions, they are aware that their self‐assessment is not correct. Hence, unattractive people may admit that they perceive their physical attractiveness in a positively biased way.

### The present research

Six studies examine the hypothesis that objectively unattractive people overestimate their attractiveness. In contrast, attractive people should have more accurate views of their attractiveness. A further goal of the present research was to illuminate why unattractive people are prone to unrealistically positive judgments of their attractiveness. In all studies, participant’s self‐perceived attractiveness was compared with how participants were rated by strangers (university students, about the same age as most of the participants). These judges' ratings were employed as a proxy for participant’s objective attractiveness. With the exception of Study 4, all studies were part of student projects that employed measures that are not relevant for the present purposes. These additional measures are not reported here, but the data are publicly available (https://osf.io/ndqcy/). Each study was run over the course of one semester, with the aim to run as many participants as possible.

## STUDY 1

Study 1 provides a first test of the hypothesis that unattractive people overestimate their attractiveness. A further aim was to investigate whether unattractive people are aware of their biased self‐perceptions.

### Method

#### Participants, measures, and procedure

Participants were 191 individuals (130 females, 61 males; mean age = 22.1 years, *SD *=* *2.5) who were approached close to the university campus and asked whether they would be willing to respond to around 100 questions concerning their personality. Among these personality questions (e.g., HEXACO, self‐esteem, narcissism), participant’s subjective attractiveness was assessed (“how physically attractive do you think you are”). Participants also indicated their belief about how physically attractive they are perceived by strangers. All items were assessed on a scale from 1 (*not at all attractive*) to 9 (*very attractive*). They then indicated their belief about how many people of the same age and gender are more physically attractive than themselves. The scale was from 0% (*I am more attractive than all others*) to 100% (*I am less attractive than all others*), in 10% increments. Finally, participants were asked whether they might perceive their physical attractiveness in a biased way, using a scale from −5 (*I perceive myself to be significantly less attractive than I actually am*) to +5 (*I perceive myself to be significantly more attractive than I actually am*) with a midpoint of 0 (*I perceive myself to be as attractive as I actually am; I am neither negatively nor positively biased*). This measure of participant’s self‐reported perceived bias was adapted from Bollich et al. (2015). Participants were then thanked and debriefed. While participants responded to the questionnaire, their objective attractiveness was unobtrusively assessed by two male experimenters who were standing in front of the participants while they were filling out the questionnaire. The experimenters independently judged how physically attractive they perceived the respondent, on a scale from 1 (*not at all attractive*) to 9 (*very attractive*). Experimenter ratings were significantly correlated, *r*(191) = 0.59, *p *<* *0.001, and were thus averaged. In this study and the following studies, raters and participants were unacquainted and raters were unaware of how participants judged themselves.

### Results

Subjective and objective attractiveness ratings were not significantly correlated, *r*(191) = 0.13, *p *=* *0.067. Participants perceived themselves to be more attractive (*M *=* *6.03, *SD *=* *1.08) compared to how they were perceived by the experimenters (*M *=* *5.44, *SD *=* *1.44), *t*(190) = 4.82, *p *<* *0.001.

To test the hypothesis that unattractive more than attractive people overestimate their attractiveness, it was examined whether the difference between the subjective and objective attractiveness ratings would differ as a function of the participant’s objective attractiveness. In fact, the correlation between the participant’s objective attractiveness and the difference between the subjective and objective attractiveness ratings was significantly negative, *r*(191) = −0.80, *p *<* *0.001. However, this analysis cannot show whether unattractive participants overestimate their attractiveness and/or attractive participants underestimate it. Hence, to illustrate the exact impact of the participant’s objective attractiveness on how the participant’s subjective attractiveness differed from the objective attractiveness ratings, participants were split into four groups based on their objective attractiveness (unattractive, below average, above average, attractive). This procedure is based on research into the Dunning‐Kruger effect that also divided participants into four groups (from bottom quartile performers up to top quartile performers) based on their objective performance (e.g., Kruger & Dunning, 1999).

**Fig. 1:**
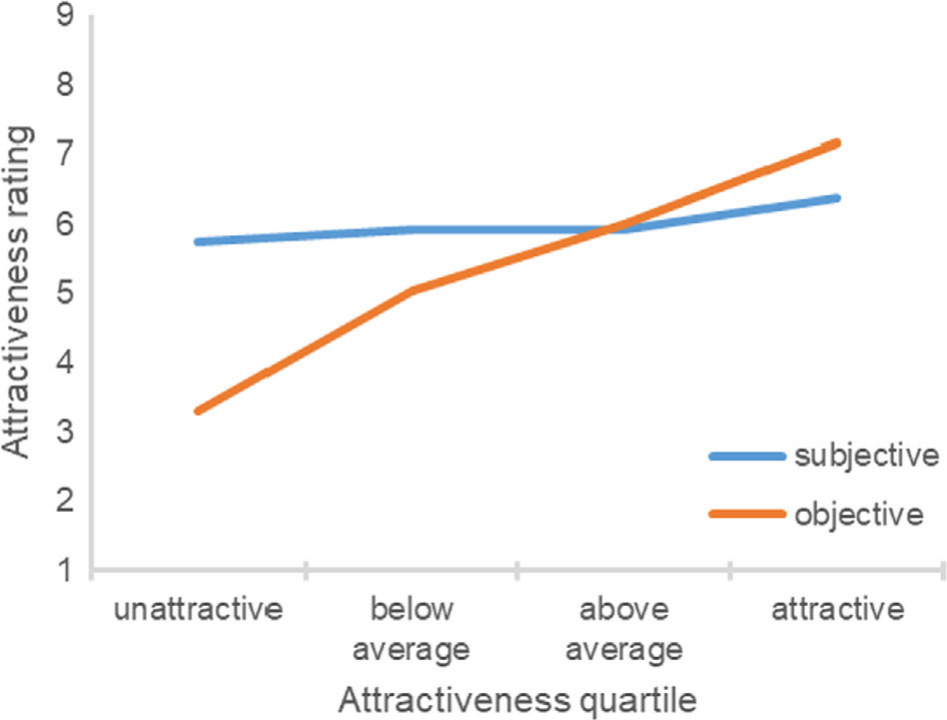
Ratings of subjective attractiveness as a function of the participant’s objective attractiveness (Study 1). [Colour figure can be viewed at wileyonlinelibrary.com]

Figure 1 shows the participant’s subjective and objective attractiveness ratings in each objective attractiveness quartile. An ANOVA yielded a significant effect of the participant’s objective attractiveness quartile, *F*(3, 187) = 55.01, η^2^ = 0.47, *p *<* *0.001. For unattractive participants, ratings of subjective attractiveness were much higher than ratings of objective attractiveness, one sample *t*(37) = 10.64, *p *<* *0.001. Relative to the scale midpoint, they gave themselves a score that was significantly higher, one sample *t*(37) = 4.59, *p *<* *0.001, whereas ratings by the experimenters were significantly below the scale midpoint, one sample *t*(37) = 15.54, *p *<* *0.001. For below average participants, ratings of subjective attractiveness were also higher than ratings of objective attractiveness, *t*(68) = 6.59, *p *<* *0.001, but the effect was less pronounced. For above average participants, ratings of subjective attractiveness were similar to the ratings of objective attractiveness, *t*(31) = 0.32, *p *=* *0.751. For attractive participants, ratings of subjective attractiveness were *lower* than ratings of objective attractiveness, *t*(51) = 4.37, *p *<* *0.001.^2^ Sex of participants did not moderate these findings, *F*(3, 183) = 0.54, η^2^ = 0.01, *p *=* *0.655. In the following studies, sex of participants also did not systematically moderate any of the main findings and is thus not considered further.

These findings provide compelling evidence that unattractive people perceive themselves to be more attractive relative to how they are perceived by others. But are they aware of others perceiving them as relatively unattractive? To address this issue, we compared the participant’s objective attractiveness ratings with the participant’s belief about how they are perceived by strangers in each objective attractiveness quartile. An ANOVA showed a significant effect of the participant’s objective attractiveness quartile, *F*(3, 187) = 47.72, η^2^ = 0.43, *p *<* *0.001. For unattractive participants, ratings by the experimenters were considerably lower than how participants believed to be perceived by strangers (*M *=* *5.50, *SD *=* *1.23), *t*(37) = 8.97, *p *<* *0.001. A similar, but less pronounced, effect was found for below average participants (*M *=* *5.72, *SD *=* *1.06), *t*(68) = 5.18, *p *<* *0.001. Above average participants were very accurate (*M *=* *6.00, *SD *=* *1.08), *t*(31) = 0.00, *p *=* *1.00. Attractive participants *underestimated* their attractiveness (*M *=* *6.21, *SD *=* *1.23), *t*(51) = 5.10, *p *<* *0.001.

Finally, we examined the extent to which unattractive participants were aware of their biased self‐perceptions. Self‐perceptions of bias did not significantly differ across the objective attractiveness quartiles, *F*(3, 187) = 1.95, η^2^ = 0.03, *p *=* *0.123. Most importantly, unattractive participants (*M *=* *−0.66, *SD *=* *1.56), one sample *t*(37) = 2.59, *p *=* *0.014, indicated that they perceived themselves to be less attractive than they actually are.

### Discussion

Study 1 provided suggestive evidence that objectively unattractive people inaccurately perceive their attractiveness. Relative to ratings by two experimenters, unattractive participants substantially overestimated their level of attractiveness. Whereas the experimenters gave the unattractive participants an attractiveness score that was below the scale midpoint, unattractive participants gave themselves a score that was significantly above. The data also suggest that unattractive people have little insight into how others perceive them. The participant’s belief about how they are perceived by strangers significantly differed from how the experimenters perceived the unattractive participants. In addition, unattractive participants indicated that they perceive themselves to be *less* attractive than they actually are.

Notably, whereas unattractive and below average participants considerably overestimated their attractiveness, above average and attractive participants did not. Attractive participants even underestimated their attractiveness. However, the magnitude of the unattractive participants’ overestimation was much more pronounced than the attractive participants’ underestimation. Overall – and in line with the typical above average effect – the participant’s self‐ratings were higher than the ratings by the experimenters.

It is remarkable that objectively attractive participants’ self‐ratings were only slighter higher than were self‐ratings of unattractive participants, although experimenter ratings of the attractive and unattractive participants were dramatically different. Self‐rated attractiveness of attractive and unattractive participants even did not differ at all when participants were explicitly asked to compare their attractiveness with others (see endnote 2).

## STUDY 2

Study 2 provides a conceptual replication of the main finding from Study 1 that unattractive people overestimate their attractiveness. Study 2 also addresses a limitation of Study 1 where participants were rated by only two experimenters as a measure of the participant’s objective attractiveness.

### Method

#### Participants, measures, and procedure

Participants were 163 students of an Austrian university as well as acquaintances of the experimenter (92 females, 71 males; mean age = 33.8 years, *SD *=* *16.3). After providing demographics and filling out measures that are not relevant for the present purpose (e.g., self‐esteem, narcissism, optimism), two photos of the participants were taken. Participants were informed how the photos would be used and asked whether they agree to participate. Eight (four females, four males) independent judges provided ratings of the attractiveness of the participants, employing the same item as in Study 1. These ratings were highly correlated (α = 0.86) and were averaged to form an index of the participant’s objective attractiveness. Participant’s subjective attractiveness was assessed as in Study 1. Participants also indicated their belief about how attractive they are perceived by strangers and reported on whether their self‐perceptions are biased. The findings were very similar to Study 1 and thus are not reported here. The same applies to the following studies. In each study, the ratings by the experimenters were much lower compared to how the unattractive participants believed to be perceived by strangers and unattractive participants reported to be negatively biased, in that they perceive themselves to be less attractive than they actually are (with the exception of Study 4 where the measure of self‐perceived bias was not assessed).

### Results

Subjective and objective attractiveness ratings were significantly correlated, *r*(163) = 0.32, *p *<* *0.001. Participants perceived themselves to be more attractive (*M *=* *5.93, *SD *=* *1.33) compared to how they were perceived by the experimenters (*M *=* *4.77, *SD *=* *1.21), *t*(162) = 9.96, *p *<* *0.001.

**Fig. 2:**
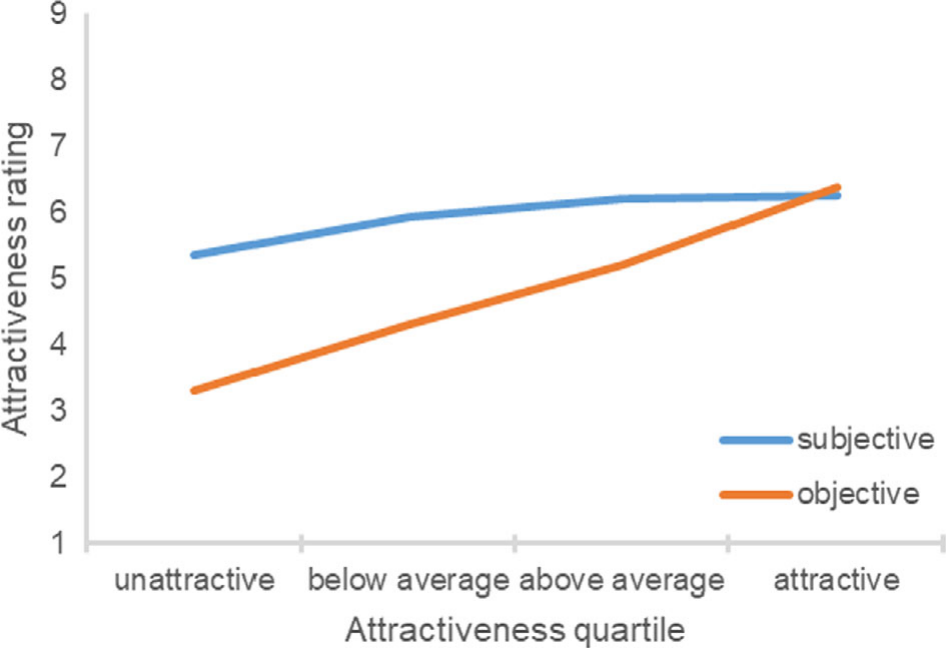
Ratings of subjective attractiveness as a function of the participant’s objective attractiveness (Study 2). [Colour figure can be viewed at wileyonlinelibrary.com]

The correlation between the participant’s objective attractiveness and the difference between the subjective and objective attractiveness ratings was significantly negative, *r*(163) = −0.53, *p *<* *0.001. Figure 2 shows the participant’s subjective and objective attractiveness ratings in each objective attractiveness quartile. An ANOVA yielded a significant effect of the participant’s objective attractiveness quartile, *F*(3, 159) = 24.10, η^2^ = 0.31, *p *<* *0.001. Unattractive, *t*(42) = 10.29, *p *<* *0.001, below average, *t*(39) = 7.40, *p *<* *0.001, and above average participants, *t*(39) = 5.34, *p *<* *0.001, perceived themselves to be more attractive compared to the ratings by the independent judges. As in Study 1, the effect was most pronounced for the unattractive participants. Attractive participants were relatively accurate, *t*(39) = 0.76, *p *=* *0.455.

### Discussion

Study 2 further showed that unattractive people overestimate their attractiveness compared to the ratings provided by the independent judges, whereas attractive people are more accurate. A limitation of Study 1 was that only two individuals provided the ratings of the participant’s objective attractiveness. Given that Study 2 employed eight judges, the measure of the participant’s objective attractiveness was likely to be more valid and it is therefore reassuring that the pattern of findings from Study 1 and 2 was very similar.

## STUDY 3

The main goal of Study 3 was to examine whether the motive to perceive oneself in a favorable light accounts for the tendency that unattractive people overestimate their attractiveness. People typically exhibit a strong tendency to discredit negative information about themselves (e.g., Ditto & Lopez, 1992; Shepperd, 1993). Empirical investigations have repeatedly shown that affirming the self‐concept may satisfy the motivation to protect one’s self‐worth and thus can counteract the biased processing of negative information about the self (Reed & Aspinwall, 1998; Sherman, Nelson & Steele, 2000). Hence, Study 3 examined whether unattractive people would be less likely to overestimate their attractiveness after an affirmation of self‐worth.

Previous research (Dunning, Meyerowitz & Holzberg, 1989) found that idiosyncratic trait definitions may contribute to self‐serving assessments. When the definition of a concept is unclear, people use these criteria that place them in the best light. For example, unattractive individuals might be aware that their body is discrepant from the ideal of beauty, but they believe they had a nice face. They then may use the attractiveness of their face as an indicator of their overall attractiveness and neglect their bodily appearance. To address the possibility that people use those attractiveness criteria that best serve their wish to be attractive, separate measures of the participant’s attractiveness of the face, body, and overall appearance were employed.

### Method

#### Participants, measures, and procedure

Participants were 235 individuals (131 females, 104 males; mean age = 22.9 years, *SD *=* *3.6). They were approached on campus and at a shopping mall. After providing demographics, participants were randomly assigned to a self‐affirmation condition or a no‐affirmation condition (adapted from Reed & Aspinwall, 1998). In the self‐affirmation condition, participants received a list of 10 kind behaviors (e.g., “Have you ever been generous and selfless to another person?”). For each behavior, they indicated whether they had ever performed the behavior, and if yes, to provide a brief written example. In the no‐affirmation condition, participants received a personal opinion survey and were asked whether they endorse each of 10 statements (e.g., “I think that the beach is a great place to vacation.”), and if yes, to provide a brief reason for their answer.

Among filler items, the participant’s subjective attractiveness was assessed with three items (α = 0.84): “How physically attractive do you think is your face?” “How physically attractive do you think is your body?” and “How physically attractive do you think is your overall appearance?” The scale was from 1 (*not at all attractive*) to 9 (*very attractive*). Unbeknownst to the participants, two female experimenters responded to the same items to assess the participant’s objective attractiveness. For both experimenters, the three items were highly correlated and thus combined. These experimenter ratings were then averaged, *r*(235) = 0.82, *p *<* *0.001.

### Results

Subjective and objective attractiveness ratings were not significantly related, *r*(235) = 0.13, *p *=* *0.052. As in Studies 1 and 2, participants perceived themselves to be more attractive (*M *=* *6.30, *SD *=* *1.18) compared to the experimenter ratings (*M *=* *5.43, *SD *=* *1.30), *t*(234) = 8.13, *p *<* *0.001.

**Fig. 3:**
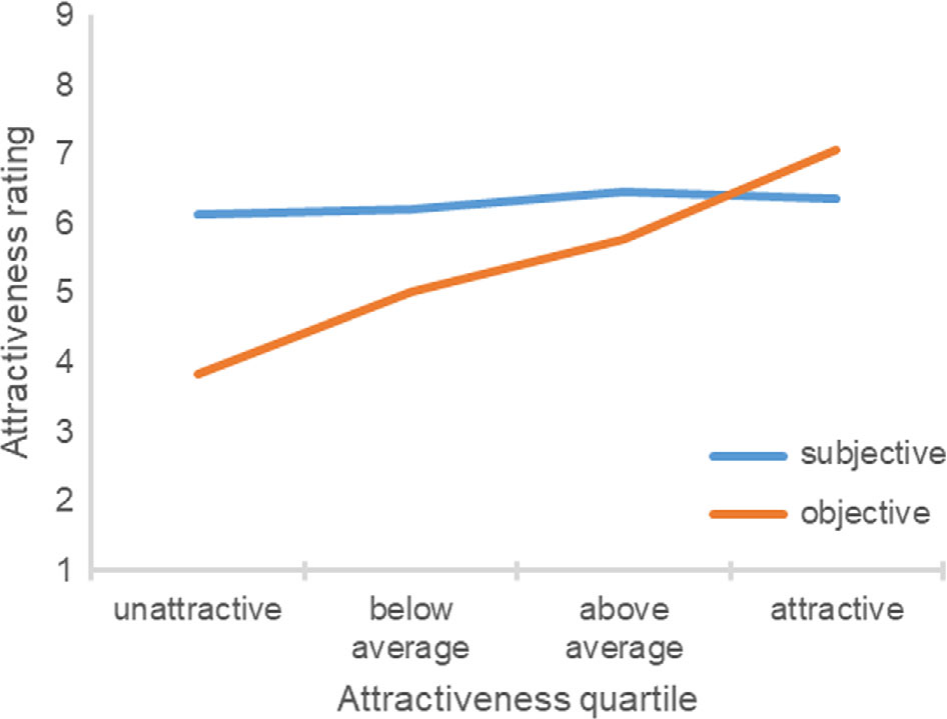
Ratings of subjective attractiveness as a function of the participant’s objective attractiveness (Study 3). [Colour figure can be viewed at wileyonlinelibrary.com]

The correlation between the participant’s objective attractiveness and the difference between the subjective and objective attractiveness ratings was significantly negative, *r*(235) = −0.70, *p *<* *0.001. Figure 3 shows the participant’s subjective and objective attractiveness ratings in each objective attractiveness quartile. An ANOVA revealed a significant effect of the participant’s objective attractiveness quartile, *F*(3, 231) = 63.30, η^2^ = 0.45, *p *<* *0.001. Unattractive, *t*(61) = 12.29, *p *<* *0.001, below average, *t*(53) = 6.61, *p *<* *0.001, and above average participants, *t*(58) = 5.45, *p *<* *0.001, perceived themselves to be more attractive compared to the experimenter ratings. In contrast, attractive participants underestimated their attractiveness, *t*(59) = 4.97, *p *<* *0.001. The pattern of findings was similar when differentiating between the attractiveness of the face, body, and overall appearance. Unattractive participants always overestimated their attractiveness.

Affirmation did not moderate the impact of the participant’s objective attractiveness on the difference between the subjective and objective attractiveness ratings, *F*(3, 227) = 0.72, η^2^ = 0.01, *p *=* *0.543, suggesting that a boosted self does not make unattractive people willing to admit that they are not attractive. In fact, ratings of subjective attractiveness by unattractive participants without (*M *=* *6.13, *SD *=* *1.38) and after engaging in self‐affirmation (*M *=* *6.15, *SD *=* *1.25) were virtually the same.

### Discussion

Study 3 replicated the main finding from Studies 1 and 2 that unattractive participants considerably overestimate their attractiveness compared to ratings by strangers. The main goal of Study 3, however, was to examine whether an affirmation of self‐worth would decrease this tendency. We employed a self‐affirmation manipulation that has successfully reduced defense biases (Reed & Aspinwall, 1998), but this manipulation had almost no effect on how unattractive participants judged their attractiveness. This (non‐significant) finding suggests that the tendency of unattractive people to overestimate their attractiveness is *not* due to defense mechanisms that protect one’s self‐worth.

## STUDY 4

Study 3 failed to show that defense processes account for the finding that unattractive people overestimate their attractiveness. Given that non‐significant effects are difficult to interpret, Study 4 provides a further test of the idea that an affirmation of self‐worth can attenuate the tendency of unattractive people to overestimate their attractiveness. Study 4 also examined a further potential mechanism. Unattractive people may mainly compare themselves to other unattractive people and hence come to the conclusion that they are not less attractive than others. In Study 4, participants were exposed to attractive or unattractive stimulus persons (or no stimuli in a control condition). It was hypothesized that unattractive participants would lower their self‐rated attractiveness (and thereby reduce the tendency to overestimate their attractiveness compared to the objective ratings) after being exposed to attractive stimulus persons (compared to the condition where they were exposed to unattractive stimulus persons or the control condition). In contrast, attractive participants should be less affected because there is little discrepancy in attractiveness between themselves and the attractive stimulus persons. Rather, it is conceivable that attractive participants enhance their self‐rated attractiveness (and thereby reduce the tendency to underestimate their attractiveness compared to the objective ratings) after being exposed to unattractive stimulus persons because it becomes obvious that they are more attractive than most others. Such a pattern of findings would provide indirect evidence for the idea that unattractive and attractive people select different persons to compare their own attractiveness to.

### Method

#### Participants, measures, and procedure

Participants were 271 individuals (153 females, 118 males; mean age = 23.0 years, *SD *=* *3.0) of a community sample. The same self‐affirmation manipulation was employed as in Study 3. Afterwards, the attractiveness of the comparison target was manipulated by exposing participants to four head and shoulders photographs in color (two females, two males) of either highly attractive or unattractive stimulus persons. The photos were relatively equal in terms of extraneous factors such as background, pose, size, brightness, contrast, distance to the target, or angle. All stimulus persons were approximately in their mid‐twenties. These photographs were successfully employed in previous research (Greitemeyer, 2007, 2010). In the control condition, participants were not exposed to any stimulus person. As a manipulation check, participants in the photographs conditions were asked about the physical attractiveness of the stimuli. The scale was from 1 (*not at all attractive*) to 9 (*very attractive*). Participant’s subjective attractiveness was assessed as in Study 1 and participants were rated by two female experimenters, *r*(270) = 0.77, *p *<* *0.001.

### Results

The attractive stimuli (*M *=* *6.15, *SD *=* *1.13) were rated as being more attractive than the unattractive stimuli (*M *=* *3.96, *SD *=* *1.19), *t*(174) = 12.40, *p *<* *0.001. Hence, the manipulation was successful. Subjective and objective attractiveness ratings were positively related, *r*(270) = 0.17, *p *=* *0.006. As in Studies 1–3, participants perceived themselves to be more attractive (*M *=* *6.28, *SD *=* *1.23) compared to the experimenter ratings (*M *=* *5.13, *SD *=* *1.77), *t*(269) = 9.52, *p *<* *0.001.

**Fig. 4:**
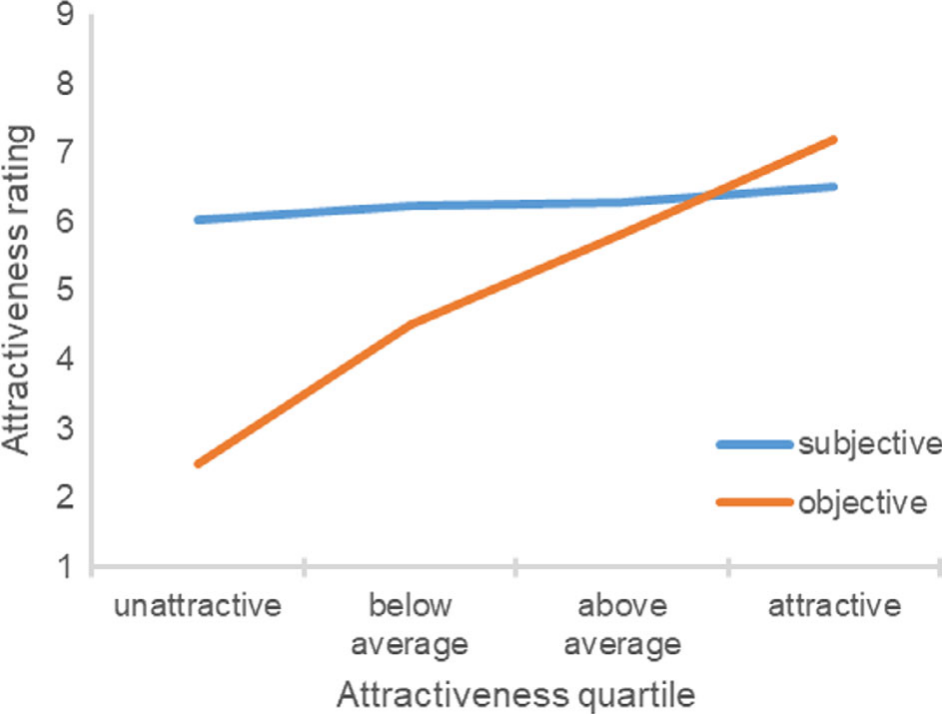
Ratings of subjective attractiveness as a function of the participant’s objective attractiveness (Study 4). [Colour figure can be viewed at wileyonlinelibrary.com]

The difference between the participant’s subjective and objective attractiveness ratings significantly differed as a function of the participant’s objective attractiveness (Fig. 4), *F*(3, 266) = 124.74, η^2^ = 0.59, *p *<* *0.001. Unattractive, *t*(55) = 17.88, *p *<* *0.001, below average, *t*(76) = 13.44, *p *<* *0.001, and above average participants, *t*(63) = 3.10, *p *=* *0.003, perceived themselves to be more attractive compared to the experimenter ratings, whereas attractive participants underestimated their attractiveness, *t*(72) = 4.24, *p *<* *0.001. The correlation between the participant’s objective attractiveness and the difference between the subjective and objective attractiveness ratings was strongly negative, *r*(270) = −0.79, *p *<* *0.001.

As in Study 3, affirmation did not moderate the impact of the participant’s objective attractiveness on the difference between the subjective and objective attractiveness ratings, *F*(3, 262) = 0.07, η^2^ = 0.00, *p *=* *0.974. Ratings of subjective attractiveness by unattractive participants after engaging in self‐affirmation (*M *=* *6.15, *SD *=* *1.58) were not lower compared to the control condition (*M *=* *5.87, *SD *=* *1.29).

Exposure to attractive/unattractive stimulus persons also did not moderate, *F*(6, 258) = 0.54, η^2^ = 0.01, *p *=* *0.779. Most importantly, unattractive participants did not lower their self‐ratings of attractiveness after being exposed to attractive stimulus persons (*M *=* *6.29, *SD *=* *1.16), compared to the exposure to unattractive stimulus persons (*M *=* *5.88, *SD *=* *1.62) or no stimuli (*M *=* *6.00, *SD *=* *1.57). Moreover, attractive participants did only slightly enhance their self‐ratings of attractiveness after being exposed to unattractive stimulus persons (*M *=* *6.66, *SD *=* *1.17), compared to the exposure to attractive stimulus persons (*M *=* *6.25, *SD *=* *1.33) or no stimuli (*M *=* *6.58, *SD *=* *1.14).

### Discussion

Study 4 once again showed that unattractive people overestimate their attractiveness. As in Study 3, an affirmation of self‐worth did not decrease this tendency. Study 4 further showed that unattractive participants were not affected by being exposed to attractive stimulus persons in the perception of their attractiveness. Taken together, neither defense mechanisms nor different comparison targets seem to account for the finding that unattractive people overestimate their attractiveness when compared to ratings by strangers. The comparison target approach could also not explain why attractive people underestimate their attractiveness. However, a more direct test of the comparison target hypothesis will be reported in Study 6.

## STUDY 5

As noted in the introduction, a lack in the metacognitive capacity to objectively assess who is competent and who is not accounts for the tendency that people of lower ability misjudge their competence (Kruger & Dunning, 1999). That is, less competent people are not only unaware that they are incompetent, but they also cannot judge how competent others are. Study 5 aims to document a similar tendency, in that unattractive people might have different beauty ideals than attractive people and thus differentiate less between attractive and unattractive stimulus persons. If unattractive people indeed have different beauty ideals, then this may explain why they overestimate their attractiveness.

### Method

#### Participants, measures, and procedure

Participants were 214 university students (151 females, 63 males; mean age = 23.1 years, *SD *=* *4.1). At the end of the survey, participants were given four photographs of attractive and four photographs of unattractive stimulus persons (two females and two males) and were asked about the physical attractiveness of each stimulus person. The scale was from 1 (*not at all attractive*) to 9 (*very attractive*). Participant’s subjective attractiveness was assessed as in Study 1 and participants were rated by two female experimenters, *r*(214) = 0.62, *p *<* *0.001.

### Results

Subjective and objective attractiveness ratings were positively related, *r*(214) = 0.18, *p *=* *0.009. As in the previous studies, participants perceived themselves to be more attractive (*M *=* *6.26, *SD *=* *1.20) compared to the experimenter ratings (*M *=* *5.69, *SD *=* *1.54), *t*(213) = 4.65, *p *<* *0.001.

**Fig. 5:**
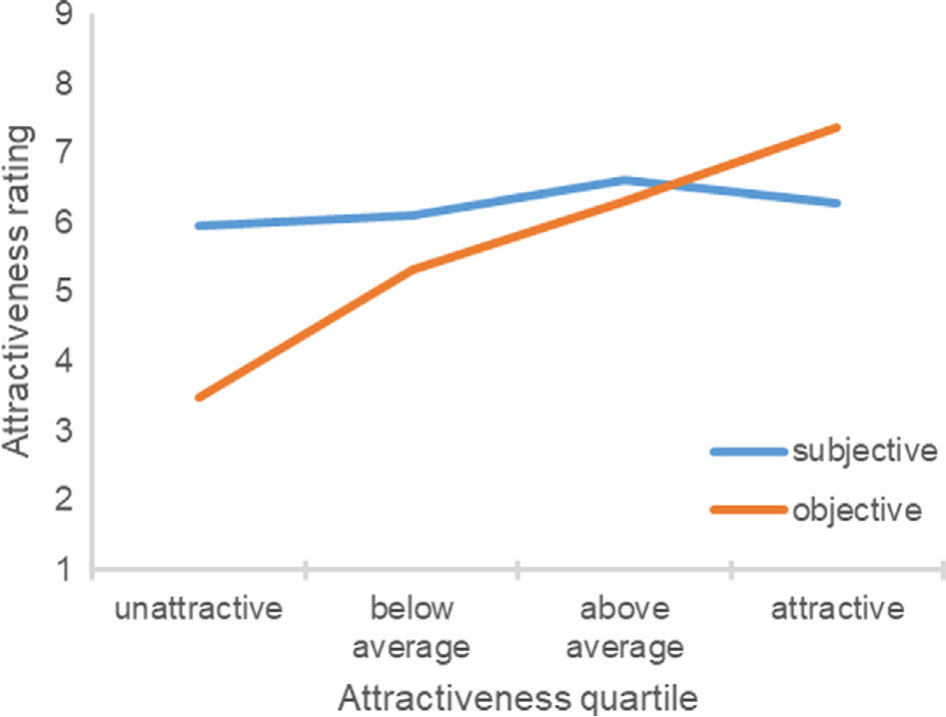
Ratings of subjective attractiveness as a function of the participant’s objective attractiveness (Study 5). [Colour figure can be viewed at wileyonlinelibrary.com]

The correlation between the participant’s objective attractiveness and the difference between the subjective and objective attractiveness ratings was strongly negative, *r*(214) = −0.75, *p *<* *0.001. The difference between the participant’s subjective and objective attractiveness ratings significantly differed as a function of the participant’s objective attractiveness (Fig. 5), *F*(3, 213) = 80.73, η^2^ = 0.54, *p *<* *0.001. Unattractive, *t*(52) = 13.16, *p *<* *0.001, and below average participants, *t*(43) = 4.50, *p *<* *0.001, overestimated their attractiveness compared to the experimenter ratings. Above average participants were relatively accurate, *t*(58) = 1.99, *p *=* *0.051. Attractive participants underestimated their attractiveness, *t*(57) = 7.31, *p *<* *0.001.

**Fig. 6:**
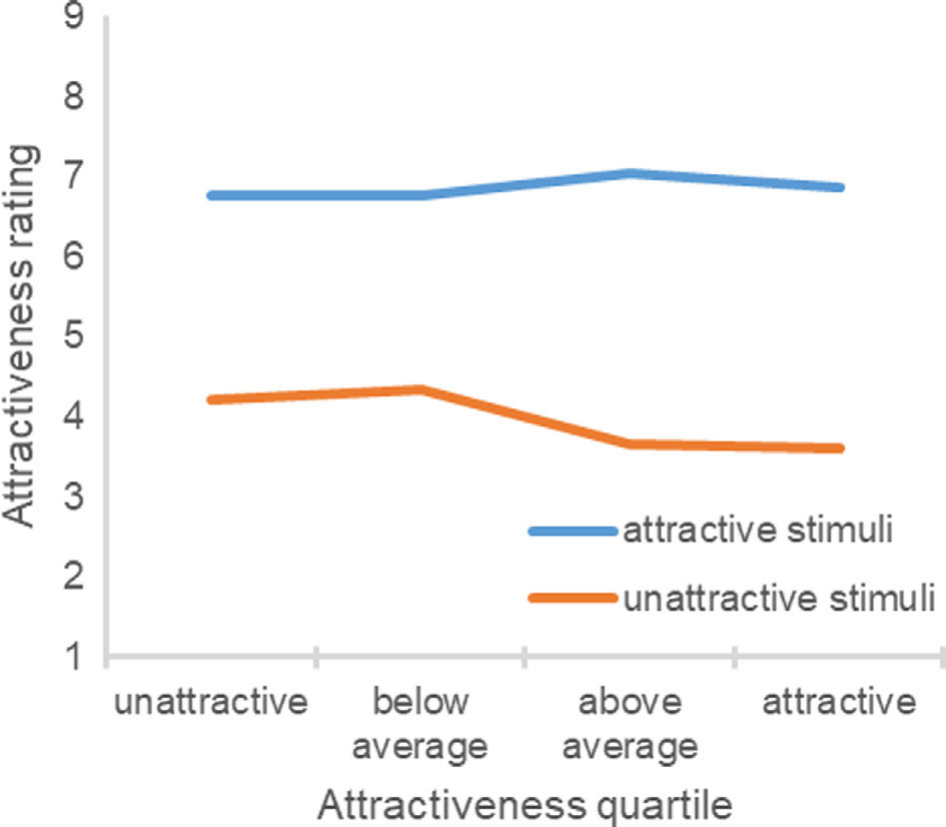
Ratings of attractive and unattractive stimuli as a function of the participant’s objective attractiveness (Study 5). [Colour figure can be viewed at wileyonlinelibrary.com]

As intended, the attractive stimuli (M = 6.86, SD = 1.15) were rated as being more attractive than the unattractive stimuli (M = 3.93, SD = 1.30), t(212) = 26.92, p < 0.001, showing that the manipulation was successful. Next, it was examined whether objectively attractive more than objectively unattractive participants would differentiate between the attractive and unattractive stimuli. In fact, the difference between the attractive and unattractive stimuli ratings significantly differed as a function of the participant’s objective attractiveness (Fig. 6), *F*(3, 209) = 5.18, η^2^ = 0.07, *p *=* *0.002. Whereas unattractive stimuli ratings significantly differed across the objective attractiveness quartiles, *F*(3, 209) = 4.50, η^2^ = 0.06, *p *=* *0.004, attractive stimuli ratings did not, *F*(3, 209) = 0.70, η^2^ = 0.01, *p *=* *0.560. The participant’s objective attractiveness was positively related to the difference between the attractive and unattractive stimuli ratings, *r*(213) = 0.18, *p *=* *0.008, negatively related to the unattractive stimuli ratings, *r*(213) = −0.17, *p *=* *0.011, and unrelated to the attractive stimuli ratings, *r*(213) = 0.05, *p *=* *0.451. Overall, attractive more than unattractive participants differentiated between attractive and unattractive individuals. In particular, unattractive participants were more favorable toward unattractive stimulus persons than were attractive participants.

However, this apparent lack in meta‐cognitive capability to differentiate between attractive and unattractive stimulus persons did not account for the effect that unattractive participants overestimated their attractiveness. The moderating impact of the participant’s objective attractiveness on the difference between the subjective and objective attractiveness ratings was unaffected by whether controlling for meta‐cognitive capability, *F*(3, 210) = 80.38, η^2^ = 0.54, *p *<* *0.001, or not, *F*(3, 213) = 80.73, η^2^ = 0.54, *p *<* *0.001. Likewise, the correlation between the participant’s objective attractiveness and the difference between the subjective and objective attractiveness ratings, *r*(214) = −0.75, *p *<* *0.001, was identical to the partial correlation when controlling for meta‐cognitive capability, *r*(210) = −0.75, *p *<* *0.001.

### Discussion

Study 5 replicated the main finding that unattractive people overestimate their attractiveness compared to ratings by strangers. Advancing the previous studies, Study 5 showed that unattractive less than attractive participants differentiate between attractive and unattractive stimulus persons. In particular, they perceived unattractive stimulus persons to be more attractive compared to how attractive participants perceived them. Hence, it would have been possible that the failure to recognize one’s own unattractiveness is due to unattractive people having different beauty ideals that are applied not only to others but also to oneself. However, this was not the case. The metacognitive capacity to recognize who is attractive and who is not did not have an impact on the participant’s ratings of their attractiveness. Because the participant’s objective attractiveness did have an impact on the participant’s beauty ideal, Study 6 provides a further test of the meta‐cognitive capacity account.

## STUDY 6

One aim of Study 6 was to examine whether unattractive more than attractive people select unattractive others to compare their attractiveness to, whereas attractive more than unattractive people select attractive others to compare their attractiveness to. If unattractive and attractive people indeed select different comparison targets, then both unattractive and attractive people may come to the conclusion that their attractiveness level is similar to others, which might explain why unattractive people overestimate their attractiveness and attractive people underestimate it. As in Study 5, it was further examined whether unattractive less than attractive people would differentiate between attractive and unattractive stimulus persons and whether such a tendency would account for the finding that unattractive people overestimate their attractiveness. Finally, to assess participant’s objective attractiveness, Study 6 employed a round robin design (Kenny, 1994) where groups of participants rate each other.

### Method

#### Participants, procedure, and measures

Participants were 106 students of an Austrian university (41 females, 65 males; mean age = 24.4 years, *SD *=* *4.1). Participants were in groups between four and 10 (mean number: 8.2). In total, 13 groups were run. Participants first responded to the same question assessing their subjective attractiveness as in Study 1. They were then asked to rate the attractiveness of the other participants, employing the same question. For each target participant, these ratings were then averaged and employed as a measure of their objective attractiveness. Afterward, participants were given photographs of four stimulus persons (one attractive female, one unattractive female, one attractive male, and one unattractive male) and asked to select one person with whom they would most likely compare their physical attractiveness to. As dependent measure, it was recorded whether participants selected an attractive or an unattractive stimulus person. On the next page of the questionnaire, photographs of the same stimulus persons were shown and participants were asked about the physical attractiveness of each stimulus person. The same measure was employed as in Study 5.

### Results

Subjective and objective attractiveness ratings were positively related, *r*(106) = 0.31, *p *=* *0.001. Participants perceived themselves to be more attractive (*M *=* *6.17, *SD *=* *1.05) compared to how they were perceived by the other participants (*M *=* *5.89, *SD *=* *1.02), *t*(105) = 2.21, *p *=* *0.030, although the effect was smaller than in Studies 1–5.

**Fig. 7:**
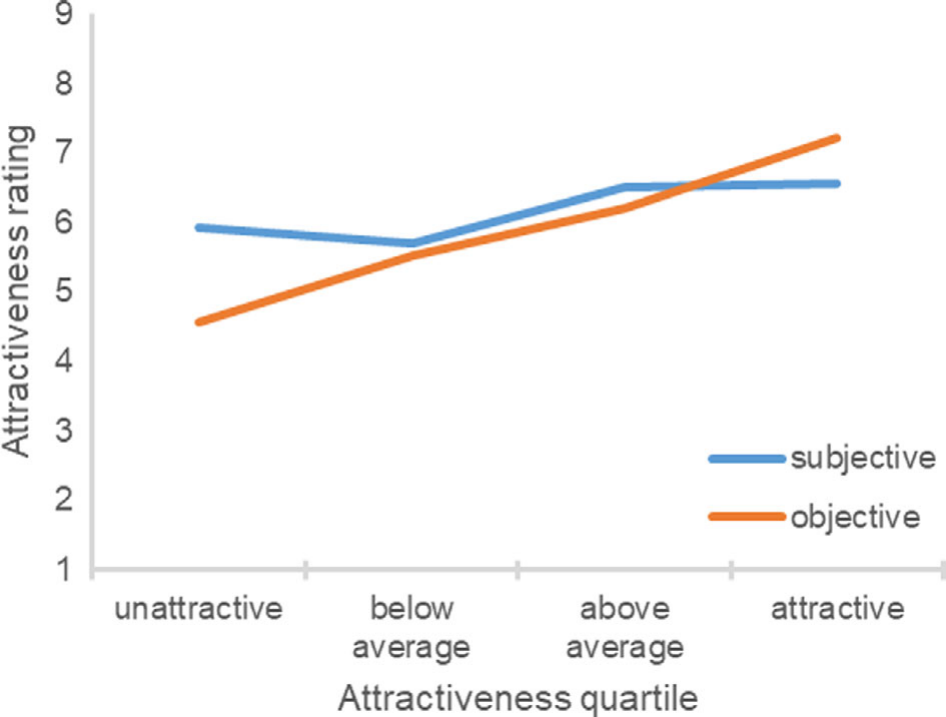
Ratings of subjective attractiveness as a function of the participant’s objective attractiveness (Study 6). [Colour figure can be viewed at wileyonlinelibrary.com]

The correlation between the participant’s objective attractiveness and the difference between the subjective and objective attractiveness ratings was negative, *r*(106) = −0.52, *p *<* *0.001. The difference between the subjective and objective attractiveness ratings significantly differed as a function of the participant’s objective attractiveness (Fig. 7), *F*(3, 102) = 14.98, η^2^ = 0.31, *p *<* *0.001. Unattractive participants overestimated their attractiveness, *t*(25) = 10.11, *p *<* *0.001. Below average, *t*(26) = 0.66, *p *=* *0.513, and above average participants, *t*(25) = 1.23, *p *=* *0.232, were relatively accurate. Attractive participants underestimated their attractiveness, *t*(26) = 3.55, *p *=* *0.001.

**Fig. 8:**
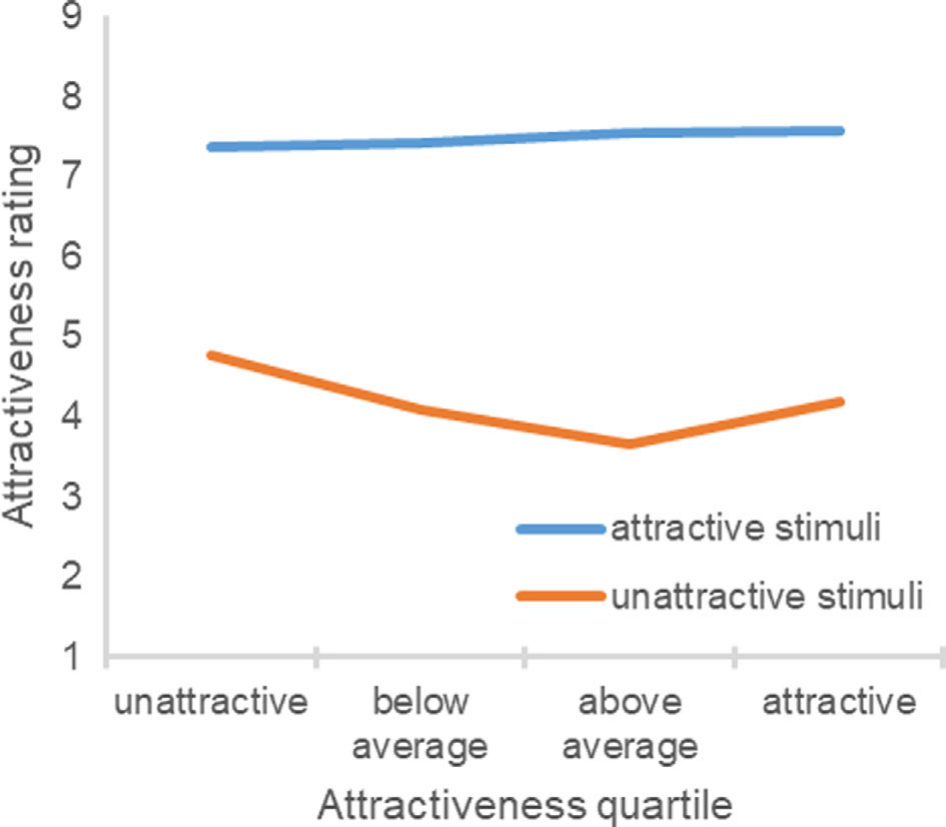
Ratings of attractive and unattractive stimuli as a function of the participant’s objective attractiveness (Study 6). [Colour figure can be viewed at wileyonlinelibrary.com]

As intended, the attractive stimuli (M = 7.50, SD = 0.83) were rated as being more attractive than the unattractive stimuli (M = 4.18, SD = 1.12), t(101) = 25.74, p < 0.001, showing that the manipulation was successful. As in Study 5, the difference between the attractive and unattractive stimuli ratings significantly differed as a function of the participant’s objective attractiveness (Fig. 8), *F*(3, 98) = 4.81, η^2^ = 0.13, *p *=* *0.004. Whereas unattractive stimuli ratings significantly differed across the objective attractiveness quartiles, *F*(3, 98) = 4.51, η^2^ = 0.12, *p *=* *0.005, attractive stimuli ratings did not, *F*(3, 102) = 0.36, η^2^ = 0.01, *p *=* *0.785. The participant’s objective attractiveness was positively related to the difference between the attractive and unattractive stimuli ratings, *r*(102) = 0.20, *p *=* *0.040. Whereas the participant’s objective attractiveness was not related to the attractive stimuli ratings, *r*(106) = 0.04, *p *=* *0.694, it was negatively related to the unattractive stimuli ratings, *r*(102) = −0.20, *p *=* *0.044.

Hence, Study 6 replicated the finding from Study 5 that unattractive people differentiate less than attractive people between attractive and unattractive stimulus persons. However, as in Study 5, meta‐cognitive capacity did not account for the finding that unattractive participants overestimate their attractiveness. When controlling for meta‐cognitive capability, the moderating impact of the participant’s objective attractiveness on the difference between the subjective and objective attractiveness ratings remained significant, *F*(3, 97) = 16.04, η^2^ = 0.33, *p *<* *0.001, and was similar to the results of the analysis without the covariate, *F*(3, 102) = 14.98, η^2^ = 0.31, *p *<* *0.001. Moreover, the partial correlation between the participant’s objective attractiveness and the difference between the subjective and objective attractiveness ratings when controlling for meta‐cognitive capability, *r*(99) = −0.54, *p *<* *0.001, was about the same as the correlation without including the covariate, *r*(106) = −0.52, *p *<* *0.001.

**Fig. 9:**
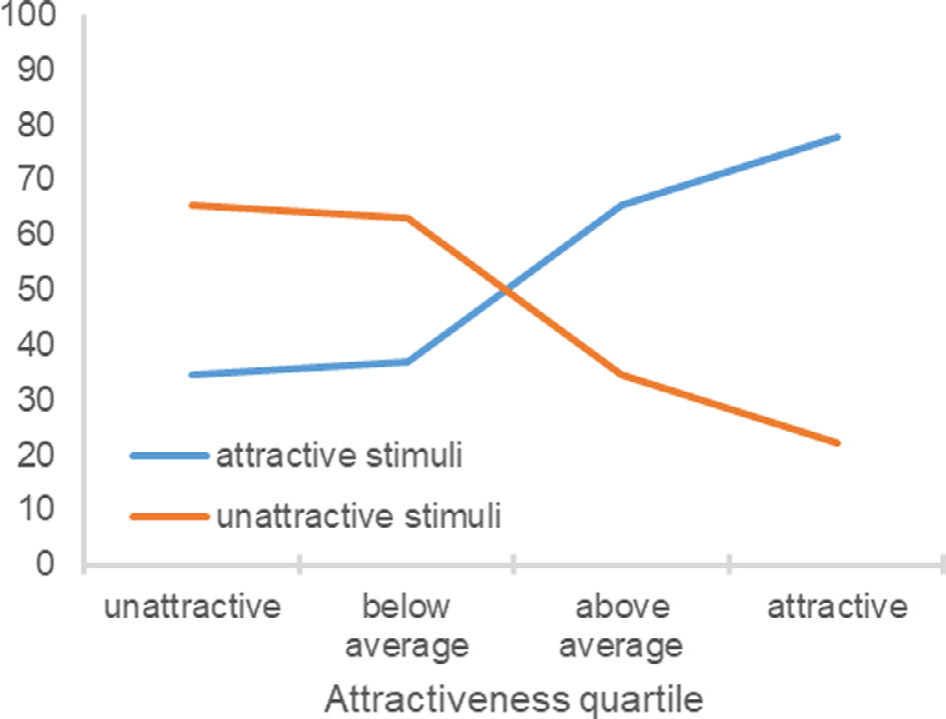
Selection (in percentage) of attractive and unattractive comparisons as a function of the participant’s objective attractiveness (Study 6). [Colour figure can be viewed at wileyonlinelibrary.com]

Next, it was examined whether unattractive participants were more likely than attractive participants to select an unattractive stimulus person with whom they would compare their attractiveness to. In fact, whereas the majority of the unattractive and the below average participants selected an unattractive comparison stimulus person, the majority of the above average and the attractive participants selected an attractive comparison stimulus person, χ^2^(3, 106) = 14.55, *p *=* *0.002 (Fig. 9).

However, the selection of different comparison targets did not moderate the impact of the participant’s objective attractiveness on the difference between the subjective and objective attractiveness ratings, *F*(3, 98) = 1.74, η^2^ = 0.05, *p *=* *0.163. Unattractive participants that compared themselves with an unattractive stimulus person (*M* = 1.21, *SD* = 0.61) did not overestimate their attractiveness more than those unattractive participants that compared themselves with an attractive stimulus person (*M* = 1.57, *SD* = 0.76). Moreover, attractive participants that compared themselves with an attractive stimulus person (*M* = −0.36, *SD* = 0.82) did not underestimate their attractiveness more than those attractive participants that compared themselves with an unattractive stimulus person (*M* = −1.69, *SD* = 0.73). If anything, opposing trends were found.

### Discussion

Study 6 replicated Study 5 that unattractive people differentiate less than attractive people between unattractive and attractive stimulus persons. In particular, unattractive participants were more favorable toward unattractive stimulus persons. Study 6 further showed that unattractive participants were more likely than attractive participants to select an unattractive stimulus person with whom they would compare their attractiveness to. However, neither of these tendencies could explain why unattractive participants overestimated their attractiveness compared to the ratings by the other participants.

## GENERAL DISCUSSION

The present set of studies addressed the relationship between self‐ratings of attractiveness and ratings by others. As in previous research (for meta‐analyses, Feingold, 1992; Langlois et al., 2000), the experimenters (Studies 1, 3–5), raters of the participant’s photographs (Study 2), and other participants (Study 6) showed high agreement about whether a person is attractive or not. In contrast, the relationship between the participant’s subjective and objective attractiveness ratings was relatively small. That is, whereas the interrater agreement of ratings of a target’s attractiveness was high, some of the targets had a different perception of how attractive they are.

All six studies provide compelling evidence that self‐ratings of unattractive people mostly differ from how others perceive their attractiveness. In fact, relative to ratings by strangers, all studies showed that unattractive participants considerably overestimated their attractiveness. It is remarkable that across all studies, unattractive participants reported to be above‐average (relative to the scale midpoint) and their self‐rated attractiveness was similar to how the objectively attractive participants rated their attractiveness. Moreover, unattractive participants were mostly unaware of how others rate their attractiveness. The objective attractiveness was much lower than how the unattractive participants believed to be perceived by strangers. Overall, unattractive participants judged themselves to be of about average attractiveness and they showed very little awareness that strangers do not share this view. In contrast, attractive participants had more insights into how attractive they actually are. If anything, they underestimated their attractiveness. It thus appears that unattractive people maintain illusory self‐perceptions of their attractiveness, whereas attractive people’s self‐views are more grounded in reality.

Whereas the effect that unattractive people overestimate their attractiveness compared to ratings by strangers could be firmly established, elucidating the exact underlying mechanisms awaits future research. The present studies tested some possible mechanisms but these appeared not to be the driving forces. Based on self‐affirmation theory (Steele, 1988), it was reasoned that if the overestimation effect has motivational roots, then affirming other aspects of the self should reduce defensive processes so that more accurate self‐perceptions result. However, both Studies 3 and 4 showed that a self‐affirmation manipulation that had successfully reduced defensive processing in previous research (Reed & Aspinwall, 1998) did not affect how unattractive participants rated their attractiveness. Hence, it appears that the wish to perceive oneself in a favorable way is not the main mechanism why unattractive people overestimate their attractiveness.

However, meta‐cognitive capacity and the comparison target approach also did not explain why the unattractive participants overestimated their attractiveness. Kruger and Dunning (1999) argued that incompetent people lack metacognitive skills that are needed to discern that one’s performance is poor. In line with their theorizing, they found that relatively incompetent participants were less able to gauge the competence of their peers than were relatively competent participants. We found a similar effect, in that unattractive participants differentiated less between attractive and unattractive stimulus persons than did attractive participants. In particular, they gave unattractive stimulus persons higher ratings than did attractive participants, whereas attractive stimulus persons were rated similarly. It thus appears that unattractive people not only perceive themselves as relatively attractive, they also rate other unattractive individuals relatively favorably. However, that unattractive people have particular beauty ideals (or have less meta‐cognitive skills to differentiate between attractive and unattractive stimulus persons) did not have an impact on how they perceive themselves. That is, that unattractive participants overestimated their attractiveness compared to ratings by strangers is not due to them rating all unattractive people (including themselves) relatively favorably.

Likewise, we did find the predicted effects that unattractive participants selected unattractive stimulus persons and attractive participants selected attractive stimulus persons with whom they would compare their attractiveness to. Hence, it would have been possible that both attractive and unattractive people believe that their attractiveness level is similar to most others, which could have explained the findings that attractive participants underestimated their attractiveness and that unattractive participants overestimated it. However, whether participants selected an attractive or unattractive stimulus person had no impact on how they rated their own attractiveness and thus could not explain why the self‐rated attractiveness of attractive and unattractive people hardly differed.

Although comparison choice did not have an impact on how unattractive participants rated their own attractiveness, the finding that unattractive participants selected unattractive stimulus persons with whom they would compare their attractiveness to suggests that they may have an inkling that they are less attractive than they want it to be. Given that people tend to compare themselves with those who they feel are similar (Wood, 1989), it appears that the unattractive participants realized that they had more in common with the unattractive rather than the attractive stimulus persons. Even though the self‐ratings of the unattractive participants suggest otherwise and that the unattractive participants reported to perceive themselves to be less attractive than they actually are, they seem to realize that they are less attractive than others.

### Limitations and future research

Whereas the finding that unattractive people overestimate their attractiveness is extremely robust and can be considered a fact (in all studies, the effect sizes were large and relatively consistent in their magnitude), the underlying mechanisms are unclear so far. Theoretical explanations are available and were tested in the present research, but although some promising effects were found (e.g., attractive and unattractive participants differed in their ratings of unattractive stimulus persons), the mechanism why unattractive people overestimate their attractiveness is still unknown and needs further work.

In this regard, it might be important that the present research compared self‐ratings of attractiveness with attractiveness ratings by strangers. Previous research has shown that self‐ratings are typically higher than ratings by strangers (a finding that was consistently replicated in the present research), but self‐ratings tend to be lower than ratings by spouses (e.g., Murstein & Christy, 1976). More generally, it has repeatedly been shown that not only objectively visible traits but also contextual variables can influence how people’s physical attractiveness is rated by others (e.g., Faust, Chatterjee & Christopoulos, 2018; Kniffin & Wilson, 2004). For example, factors unrelated to physical features such as membership in a common social group (Escasa, Gray & Patton, 2010) or feelings toward other people (Kniffin, Wansink, Griskevicius & Wilson, 2014) have been shown to have an impact on the perception of others' attractiveness. In sum, there is the strong tendency that people rate a familiar individual that they also like as more attractive than would someone who is unfamiliar with that individual. As a consequence, it may well be that there is more concordance between how unattractive people perceive themselves and how they are perceived by others with whom they have social ties.

Moreover, whereas there is generally high agreement about who is attractive and who is not, beauty is still to some extent in the eye of the beholder. For example, in one study (Cross & Cross, 1971), 300 judges rated the attractiveness of stimulus persons in groups of six. The most attractive person was picked as best of its group by 207 judges, but even the least attractive person was chosen as best of its group by four judges. Interestingly, whereas there is relatively high agreement about the attractiveness of very attractive, attractive, about average, and unattractive individuals, there is rather disagreement about who is very unattractive (Kanazawa, Hu & Larere, 2018), meaning that very unattractive individuals are attractive to some (as in the Cross & Cross, 1971, study).

It thus may be that unattractive people take the positive feedback from their loved ones and those (few) that are attracted to them and use these as anchors for their self‐ratings and for how they believe they are rated by strangers. In fact, people selectively forget (Sedikides, Green, Saunders, Skowronksi & Zengel, 2016) and denigrate (Ditto & Lopez, 1992; Shepperd, 1993) negative feedback about themselves and they preferentially want to receive self‐enhancing feedback (Gaertner, Sedikides & Cai, 2012). Future research would be thus welcome that assesses the objective attractiveness of a target by raters that know the attractiveness target and examines to what extent people integrate these ratings into their self‐perceived attractiveness.

There is a further reason why a comparison between self‐ratings and ratings by people who are familiar with the target person would be worthwhile. As the present studies suggest, the unattractive participants deceived themselves in that they perceived themselves as more attractive than is actually warranted. Evolutionary theorizing (e.g., von Hippel & Trivers, 2011) argues that such an instance of overconfidence may have social advantages. For example, people may hold inflated self‐views as a means of persuading others to adopt these overly positive perceptions of them. That is, self‐deception evolved because it facilitates the deception of others. In line with these ideas, recent research (Murphy, von Hippel, Dubbs* et al*., 2015) tested whether overconfidence is associated with desirableness as a dating partner. In fact, overconfident authors’ of dating profiles were perceived as more desirable and this effect was mediated by how confident raters perceived the authors to be. Therefore, it might be that people who learn that an objectively unattractive individual perceives him/herself in a positive way may assume that this person has some physical qualities that warrant the confidence and, in turn, perceive the person more favorably. Future research may examine whether self‐ratings of attractiveness indeed have an impact on how an individual is perceived by others after the raters learned about the self‐ratings.

Another avenue for future research would be to examine why attractive participants underestimated how attractive they were rated by strangers. Part of the reason could be due to regression‐to‐the‐mean (if participants are rated very highly by others, there is little room left for overestimation). However, the unattractive participants’ overestimation was much more pronounced than was the attractive participants’ underestimation so the finding that attractive people underestimate their attractiveness is likely to have psychological roots as well.

Finally, a limitation of the present studies is that the raters in all studies could only see the faces and clothed bodies of the participants. It is rather likely that the participants considered not only their faces and dressed appearance but also their naked bodies to estimate their level of attractiveness. Hence, part of the reason why the self‐ratings of unattractive participants differed from the experimenter ratings could be that different criteria were used as indicators of the overall attractiveness (cf., Dunning et al., 1989).

### Conclusion

Most people agree about who is attractive and who is not and attractive people are mostly aware of their level of attractiveness. As Marcus and Miller (2003, p. 334) put it: “we know who is pretty or handsome, and those who are attractive know it as well.” However, it appears that those who are unattractive do not know that they are unattractive.

I am grateful to Martin Bayer, Vanessa Deubzer, Boris Duspara, Christoph Herz, Nicola Hutzenthaler, Johanna Kießling, Nora Peglow, Annika Rellig, Carina Röckel, and Janny Sauter for their help in conducting this research.

## Notes

^1^ In the following, for the sake of brevity, the term attractiveness is used to denote a person’s physical attractiveness.

^2^ Participants’ subjective attractive ratings were remarkably similar across the objective attractiveness quartiles, *F*(3, 187) = 2.94, η^2^ = 0.05, *p *=* *0.034. A similar pattern occurred when participants were asked to compare themselves to others, *F*(3, 187) = 1.91, η^2^ = 0.03, *p *=* *0.129.
